# Loiasis in US Traveler Returning from Bioko Island, Equatorial Guinea, 2016 

**DOI:** 10.3201/eid2301.161427

**Published:** 2017-01

**Authors:** David H. Priest, Thomas B. Nutman

**Affiliations:** Novant Health, Winston-Salem, North Carolina, USA (D.H. Priest);; National Institute of Allergy and Infectious Diseases, National Institutes of Health, Bethesda, Maryland, USA (T.B. Nutman)

**Keywords:** Loa loa, loiasis, Chrysops species, Chrysops files, deerflies, Chrysops dimidiate, Chrysops silacea, Onchocerca volvulus, Wuchereria bancrofti, parasites, filarial nematode, African eye worm, onchocerciasis, lymphatic filariasis, neglected tropical diseases, Bioko Island, Equatorial Guinea, traveler, travel medicine, filarial infections, vector-borne infections, autochthonous infection, diethylcarbamazine, ivermectin

## Abstract

The filarial parasite *Loa loa* overlaps geographically with *Onchocera volvul*us and *Wuchereria bancrofti* filariae in central Africa. Accurate information regarding this overlap is critical to elimination programs targeting *O. volvulus* and *W. bancrofti*. We describe a case of loiasis in a traveler returning from Bioko Island, Equatorial Guinea, a location heretofore unknown for *L. loa* transmission.

Loiasis (African eye worm disease) is caused by infection with *Loa loa*, a parasitic vector-borne filarial worm endemic to 10 countries in central and western Africa, including Equatorial Guinea (*1*). The worm, spread by the bite of *Chrysops* *dimidiata* and *C. silacea* flies, is of public health concern because of its geographic overlap with *Onchocerca volvulus* and *Wuchereria bancrofti* worms, which cause onchocerciasis and lymphatic filariasis, respectively (*2*). Mass drug administration programs for onchocerciasis and lymphatic filariasis often include ivermectin, which can cause serious and occasionally fatal adverse neurologic reactions in persons with high levels of circulating *L. loa* microfilariae (*3*). To avoid such reactions, an accurate picture of the geographic distribution of *L. loa* infection is needed. Given the importance of epidemiologic data in the management of filarial infections, we report a case of loiasis in a US woman who had traveled to Equatorial Guinea.

In May 2016, a 25-year-old woman sought care in Winston-Salem, North Carolina, USA, for fatigue, swelling of her left ankle, right knee pain, and intensely pruritic skin lesions on her lower extremities. She had lived on Bioko Island, Equatorial Guinea, during October 2015–March 2016 while studying local wildlife. On Bioko Island, she frequented local water sources to bathe and wash clothes and consistently took atovaquone/proguanil for malaria prophylaxis. She did not spend time on Equatorial Guinea’s mainland or travel to other nations in central or western Africa. Her flight from the United States to Bioko Island connected in Ethiopia; she did not leave the airport. 

Symptoms developed soon after her return to North Carolina in late March 2016. Laboratory evaluations performed at that time showed a leukocyte count of 8.5 × 10^3^ cells/µL (reference range 3.4–10.8 × 10^3^ cells/µL), hemoglobin level of 13.9 (reference range 11.1–15.9 g/dL), platelet count of 219 (reference range 150–379 × 10^3^ cells /µL), and absolute eosinophil count of 2,300/µL (reference range 40–400/µL).

In May, her physical examination was notable only for edema of the left lower extremity adjacent to her ankle. Three separate midday blood smears for microfilariae were negative. Laboratory tests showed a leukocyte count of 11.5 × 10^3^ cells/µL, absolute eosinophil count of 4,200/µL, and IgE level of 175 IU/mL (reference range 0–100 IU/mL). Results of antifilarial IgG4 and *Strongyloides* IgG tests (performed by LabCorp, Burlington, NC, USA) were negative.

Over the subsequent 4 weeks, new pruritic, erythematous plaques appeared on her right flank and left thigh and behind her left ear (Figure). Blood testing at the Laboratory of Parasitic Diseases, National Institute of Allergy and Infectious Diseases, National Institutes of Health (Bethesda, MD, USA), showed negative results for a 1-mL Nuclepore (Whatman GE Lifesciences, Pittsburgh, PA, USA) filtration for microfilariae; *L. loa*–specific PCR (*4*); and rapid diagnostic testing, using the SD BIOLINE Oncho/LF IgG_4_ biplex test (Standard Diagnostics, Inc., Seoul, South Korea) for detection of specific antibodies against *O. volvulus* and *W. bancrofti*. Testing also showed a BmA IgG (*5*) level of 100.6 µg/mL (reference value <14.0 µg/mL); a normal BmA IgG4 antibody level; and a luciferase immunoprecipitation systems assay result of 456,969 light units (LU)/mL for LL-SXP1 IgG (negative value <3,000 LU/mL) and 19,193 LU/mL for LL-SXP1 IgG4 (negative value <1,700 LU/mL) (*6*).

**Figure:**
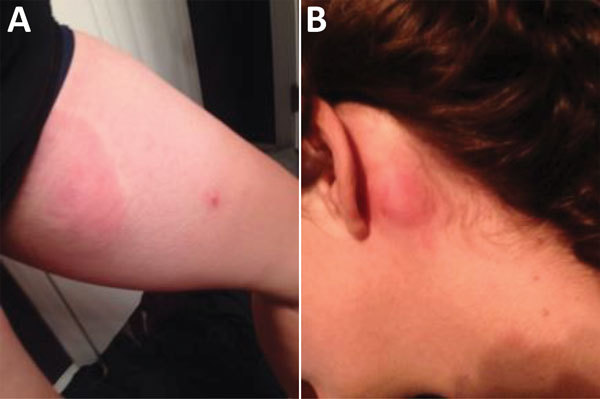
Cutaneous manifestations of *Loa loa* (African eye worm) infection in a US traveler who returned from a 6-month stay on Bioko Island, Equatorial Guinea, 2016. Urticarial lesions on the left thigh showing a coincident papular eruption (A) and behind the left ear (B).

The patient was treated with diethylcarbamazine for 21 days. After completion of treatment, her symptoms improved, and her leukocyte and eosinophil counts returned to within reference ranges.

Recent years have seen renewed interest in the epidemiology and geographic distribution of *L. loa* in central and western Africa because of the risk of encephalopathy in patients given ivermectin as part of large programs to control filarial infections. Although the intermediate hosts of *L. loa* are present on Bioko Island, previous loiasis cases were reported only in persons who had been exposed to *Chrysops* flies on mainland Africa (*7*). Given the presence of these vectors on Bioko Island and the patient’s lack of exposure to any other *L. loa*–endemic region, transmission of *L. loa* on Bioko Island seems probable. Of note, a previous study found 1 of 541 skin snips tested on Bioko Island to be PCR-positive for *L. loa*, a finding thought to have been caused by skin snip sample contamination with capillary blood (*8*).

The signs and symptoms of *L. loa* infection exhibited by the US patient reinforce the perception that loiasis in returned travelers is often quite distinct from that in persons with lifelong exposure in a region where the disease is endemic (*9*,*10*). The course of infection also points to differences in IgG- and IgG4-based antifilarial serologic testing early in infection (*5*) and provide evidence that the use of species-specific recombinant antigens can more accurately help with specific parasite diagnosis (*6*).

Knowledge of the geographic distribution of *L. loa* infection is critical because loiasis overlaps with other filarial diseases, such as onchocerciasis and lymphatic filariasis. The intermediate vectors responsible for *L. loa* transmission, *Chrysops* flies, are known to live on Bioko Island; the case we present suggests that local transmission of *L. loa* and prevalence of loiasis on the island may be higher than previously thought.
